# Correlation Analysis of Erectile Dysfunction with Lower Urinary Tract Symptoms (LUTS) Degree and Clinical Features in LUTS Patients

**Published:** 2018-05

**Authors:** Lei WANG, Tongqing WANG, Jian LIU, Jizheng WANG

**Affiliations:** Dept. of Urology, Ward 1, Zhengzhou Central Hospital Affiliated to Zhengzhou University, Zhengzhou 450007, Henan Province, China

**Keywords:** Lower urinary tract symptoms, Erectile dysfunction, Clinical features

## Abstract

**Background:**

We aimed to investigate the prevalence of erectile dysfunction (ED) in patients with lower urinary tract symptoms (LUTS) and to explore the correlation of ED with LUTS and its clinical features.

**Methods:**

Overall, 400 outpatients and inpatients with LUTS diagnosed in Zhengzhou Central Hospital, Zhengzhou University, Zhengzhou, Henan Province, China from June 2015 to June 2017 were collected. LUTS degree was assessed by the International Prostate Symptom Score (IPSS), and ED degree was assessed by the International Index of Erectile Function (IIEF-5). The correlation of ED with LUTS degree, age, blood lipids, homocysteine (Hcy) and other factors was analyzed.

**Results:**

The prevalence of ED in patients with LUTS was 82.25%. With the increase in age, the prevalence of ED was significantly elevated, and ED degree gradually became higher (*P*<0.01). The higher the degree of LUTS was, the higher the prevalence of ED would be. The degree of ED was highly correlated with the combination with hypertension, diabetes mellitus and coronary heart disease (*P*<0.05). The prevalence of ED was positively correlated with LUTS degree and the levels of total cholesterol (TC), low-density lipoprotein cholesterol (LDL-C) and Hcy (*P*<0.05 or *P*<0.01).

**Conclusion:**

ED prevalence of patients with LUTS is high, and ED is significantly related to LUTS degree, age and the levels of TC, LDL-C and Hcy in patients with LUTS.

## Introduction

Lower urinary tract symptoms (LUTS) are a common group of clinical syndromes. According to different occurrence periods, International Continence Society divided the main manifestations of LUTS into: 1) storage symptoms mainly manifested as urinary frequency, urinary urgency, urinary incontinence and nocturia; 2) voiding symptoms mainly manifested as urinary retention, dysuria, weak stream of urine, urinary bifurcation, acraturesis; 3) postmicturition symptoms mainly manifested as terminal dribbling and incomplete voiding (1).

Among the middle-aged and aged men, benign prostatic hyperplasia is the main clinical cause of LUTS (2). An epidemiological survey of the Norwegian population showed that the incidence rate of LUTS is 15.8%, in which mild LUTS accounts for 84.1%, moderate LUTS accounts for 13.2%, and severe LUTS accounts for 2.6%. Meanwhile, the occurrence rate of LUTS is gradually increased with the increase in age of patients (3).

Erectile dysfunction (ED) refers to the inability to keep an erection firm enough for 3 months or above to have a satisfactory sexual intercourse (4). The incidence rate of ED is increased with the increase in age (5, 6). In recent years, with the aging of the population in China, the incidence rate of ED has been increased year by year. About 5%–20% of men suffer from moderate to severe ED (7), which lowers the self-esteem of male patients and triggers depression and anxiety, thus seriously affecting their quality of life (8). ED is closely related to a series of risk factors such as age, obesity, hyperlipidemia, hypertension, atherosclerosis, education level, smoking, alcoholism and metabolic syndrome (9–11). The logistic regression analysis revealed that LUTS and age are independent risk factors for ED, and the risk of them is higher than that of hypertension, hyperlipidemia, diabetes mellitus and heart disease (12).

In this study, middle-aged and aged men older than 50 yr old were collected, the incidence rate of ED under different LUTS degrees was analyzed, and the relationship of ED with LUTS, age, past histories, blood lipids and serum homocysteine (Hcy) was explored.

## Methods

### Study objects

A total of 400 middle-aged and aged patients with LUTS treated or hospitalized in the Urinary Surgery Department of Zhengzhou Central Hospital Affiliated to Zhengzhou University from June 2015 to June 2017 were collected. All the included patients were aged 50–80 y old with a fixed partner, and all of them signed the informed consent.

The survey was approved by the Ethics Committee of the hospital. Exclusion criteria: 1) patients with the past history of abdominal surgery, prostate surgery or pelvic surgery; 2) patients definitely diagnosed with LUTS; 3) patients with obvious neurological diseases; 4) patients with mental disease; 5) patients suffering from urinary tract infection within the latest 1 month; 6) patients with genitourinary system hypoplasia or deformity.

### Study methods

Age, weight, height, past histories, blood lipid, Hcy level and data of other medical histories were collected.

All the surveyed people received the standardized training, and a unified survey method was used for questionnaires. LUTS scores of patients were assessed using the International Prostate Symptom Score (IPSS) (13). IPSS includes scores on urinary frequency, urinary urgency, weak stream of urine, nocturia and other 3 symptoms. Each symptom score was divided into 0–5 points (35 points in total) according to its degree. According to the score results, the total score ranging from 1 to 7 points represented mild symptoms, from 8 to 19 points represented moderate symptoms, from 20 to 35 points represented severe symptoms. The erectile function of patients was assessed using the International Index of Erectile Function (IIEF-5) (14). Patients reviewed the erectile function within recent six months, and a total of 5 symptom scores were set, each of which was divided into 0–5 points (25 points in total). According to the score results, the total score ranging from 22 to 25 points represented normal erectile function, from 12 to 21 points represented mild ED, from 8 to 11 points represented moderate ED and ≤ 7 points represented severe ED. Data of all the survey items were digitally coded. After the data were verified, two people input the data through computers to ensure the integrity and reliability of the survey data.

### Statistical methods

Statistical analysis was performed using Statistical Product and Service Solutions (SPSS) 19.0 software [International Business Machines Corporation (IBM), New York, USA]. Measurement data were expressed as *x̄*±s. The independent-samples *t* test was used for intergroup comparisons, and the one-way analysis of variance was used for comparisons among multiple groups. Comparisons of the composition ratio and percentage among multiple groups were detected using the χ^2^ test. Correlation between the relevant factors and ED was analyzed using Spearman correlation analysis. *p*<0.05 represented that the difference was statistically significant.

## Results

### ED prevalence of patients with LUTS

The total prevalence of ED in patients with LUTS was 82.25% (329/400). The mean age of the patients was (62.41±8.63) yr old. The prevalence of ED in various age groups: 70.49% (129/183) in patients aged 50–59 yr old; 88.55% (116/131) in those aged 60–69 yr old; 97.67% (84/86) in those aged over 70 yr old. With the increase in age, the prevalence of ED was significantly increased, and ED degree was gradually elevated. The differences were statistically significant (*P*=0.000) (Table 1).

**Table 1:** ED prevalence of patients with LUTS

***Age(yr)***	***Case (n)***	***Total ED prevalence***	***ED degree***			***χ_1_^2^***	***p_1_***	***χ_2_^2^***	***p_2_***
			***Mild***	***Moderate***	***Severe***				
50–59	183	129 (70.49%)	76 (41.53%)	45 (24.59%)	8 (4.37%)				
60–69	131	116 (88.55%)	39 (29.77%)	58 (44.27%)	19 (14.50%)	17.69	0.00		
≥70	86	84 (97.67%)	12 (13.95%)	25 (29.07%)	47 (54.65%)	78.73	0.00	35.66	0.00

Note: Compared with that in the group aged 50–59 years old, χ_1_^2^ is calculated; compared with that in the group aged 60–69 years old, χ_2_^2^ is calculated

### Comparisons of IPSS and IIEF-5 scores in various age groups

**Fig. 1:**
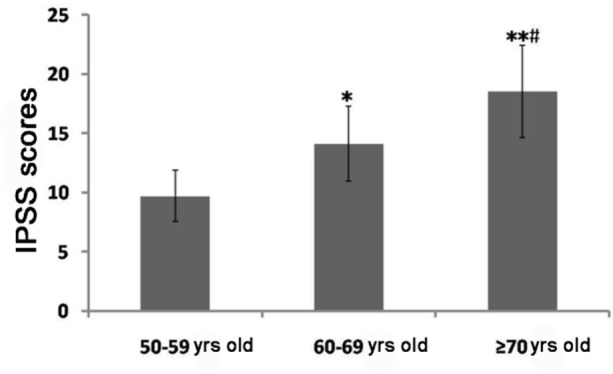
Comparisons of IPSS among various age groups The results show that IPSS is significantly increased with the increase in age Note: Compared with that in the group aged 50–59 years old, ^*^P<0.05, ^**^P<0.01; compared with that in the group aged 60–69 years old, ^#^*P*<0.05

**Fig. 2:**
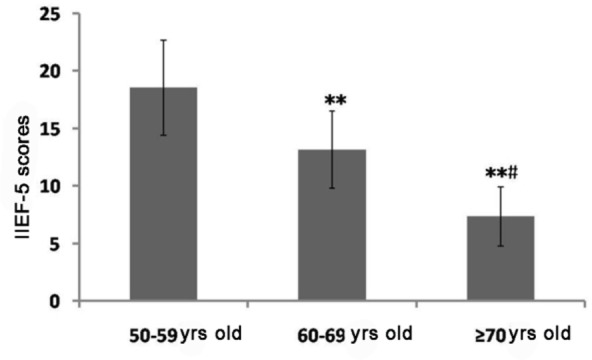
Comparisons of IIEF-5 score among various age groups The results show that IIEF-5 score is significantly decreased with the increase in age (*P*<0.05 or *P*<0.01). Note: Compared with that in the group aged 50–59 years old, ^**^P<0.01; compared with that in the group aged 60–69 years old, ^#^*P*<0.05

The average IPSS of the surveyed patient with LUTS was (14.11±3.07), and the average IIEF-5 score was (13.01±3.36). Among them, patients with mild LUTS accounted for 54.00% (n=216), those with moderate LUTS took up 35.50% (n=142) and those with severe LUTS accounted for 10.50% (n=42). Patients with normal erectile function accounted for 17.75% (n=71), those with mild ED accounted for 31.75% (n=127), those with moderate ED took up 32.00% (n=128), and those with severe ED accounted for 18.50% (n=74). There were significant differences in IPSS and IIEF-5 scores among various age groups, and the differences were statistically significant (*P*<0.05 or *P*<0.01) (Fig. 1 and 2).

### ED prevalence of patients with different degrees of LUTS

The prevalence of ED was 76.39% in patients with mild LUTS, 87.32% in patients with moderate LUTS and 95.24% in patients with severe LUTS, indicating that the higher the degree of LUTS is, the higher the prevalence of ED in patients will be, and the higher the degree of ED will be. Comparisons of ED degree among 3 groups showed that the differences were statistically significant (*P*<0.05) (Table 2).

**Table 2:** Comparisons of ED prevalence in patients with different degrees of LUTS

***LUTS***	***Case (n)***	***Total ED prevalence***	***ED degree***			***χ_1_^2^***	***p_1_***	***χ_2_^2^***	***p_2_***
			***Mild***	***Moderate***	***Severe***				
Mild	216	165 (76.39%)	88 (40.74%)	62 (28.70%)	15 (6.94%)				
Moderate	142	124 (87.32%)	32 (22.54%)	54 (38.03%)	38 (26.76%)	32.21	0.00		
Severe	42	40 (95.24%)	7 (16.67%)	12 (28.57%)	21 (50%)	37.88	0.00	6.10	0.47

Note: Compared with that in patients with mild LUTS, χ_1_^2^ is calculated; compared with that in patients with moderate LUTS, χ_2_^2^ is calculated

### Analyses of past histories of patients with different degrees of ED

The degree of ED was significantly correlated with hypertension, diabetes mellitus and coronary heart disease, and the differences were statistically significant (*P*<0.05). There were no significant differences in the distribution of obesity among ED patients (Table 3).

**Table 3:** Analyses of histories of patients with different degrees of ED

***History***	***Case [n (%)]***	***ED degree***			***χ^2^***	***P***
		***Mild***	***Moderate***	***Severe***		
Hypertension	69 (20.97%)	20 (28.99%)	25 (36.23%)	24 (34.78%)	7.66	0.02
Non-hypertension	260 (79.03%)	107 (41.15%)	103(39.62%)	50 (19.23%)		
Diabetes mellitus	75 (22.80%)	23 (30.67%)	27 (36.00%)	25 (33.33%)	6.51	0.04
Non-diabetes mellitus	254 (77.20%)	104 (40.94%)	101(39.76%)	49 (19.29%)		
Obesity	43 (13.07%)	14 (32.56%)	17 (39.53%)	12 (27.91%)	1.10	0.58
Non-obesity	286 (86.93)	113 (39.51%)	111(38.81%)	62 (21.68%)		
Coronary heart disease	57 (17.33%)	12 (21.05%)	21(36.84%)	24 (42.11%)	16.37	0.00
Non-coronary heart disease	272 (82.67)	115 (42.28%)	107(39.34%)	50 (18.38%)		

### The level of blood lipids of patients with different degrees of ED

The levels of total cholesterol (TC) and low density lipoprotein cholesterol (LDL-C) in patients with severe ED were significantly higher than those in patients with mild and moderate ED, and the differences were statistically significant (*P*<0.05). There were no significant differences in triglyceride (TG) and high-density lipoprotein cholesterol (HDL-C) among patients with different degrees of ED (Table 4).

**Table 4:** Comparisons of the level of blood lipids in patients with different degrees of ED

***Blood lipid***	***ED degree***		
	***Mild (n=127)***	***Moderate (n=128)***	***Severe (n=74)***
TG (mmol/L)	1.98±0.77	2.20±0.83	2.32±0.81
TC (mmol/L)	4.86±1.21	5.03±0.97	6.05±1.05^**#^
LDL-C (mmol/L)	2.19±0.65	2.51±0.58	3.13±0.74^**#^
HDL-C (mmol/L)	1.27±0.30	1.21±0.25	1.32±0.38

Note: Compared with that in patients with mild ED,

** *P*<0.01; compared with that in patients with moderate ED,

# *P*<0.05

### The level of serum Hcy in patients with different degrees of ED

**Fig. 3:**
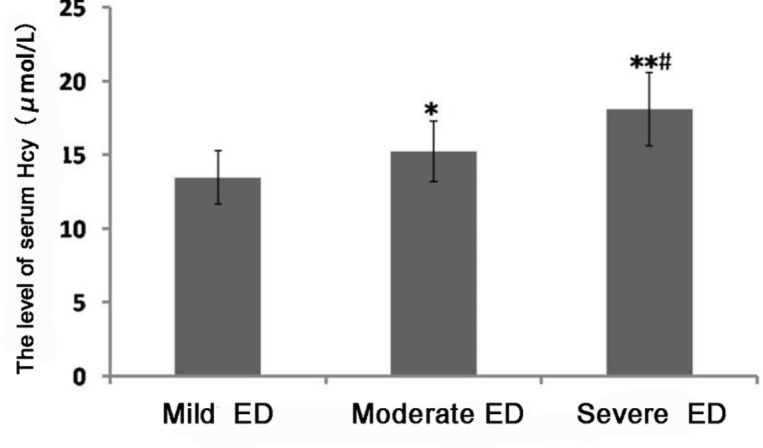
Comparisons of the level of serum Hcy in patients with different degrees of ED The results show that the level of serum Hcy is significantly elevated with the increase in ED degree (^*^P<0.05 or ^**^P<0.01). Note: Compared with that in patients with mild ED, ^*^P<0.05, ^**^P<0.01; compared with that in patients with moderate ED, ^#^*P*<0.05

Compared with that in patients with mild ED, the level of serum Hcy was significantly increased in patients with moderate and severe ED, and the differences were statistically significant (*P*<0.001 or *P*<0.01). The level of serum Hcy in patients with severe ED were significantly higher than that in patients with moderate ED (*P*<0.05) (Fig. 3).

### Correlation analyses of ED with LUTS, blood lipids and Hcy

The correlation of ED prevalence with LUTS, blood lipids and Hcy was analyzed. The results revealed that ED prevalence was positively correlated with LUTS degree and the levels of TC, LDL-C and Hcy (*P*<0.05 or *P*<0.01) (Table 5).

**Table 5:** Correlation analyses of ED with LUTS, blood lipids and Hcy

	***LUTS***	***TG***	***TC***	***LDL-C***	***HDL-C***	***Hcy***
Correlation coefficient	0.536^**^	0.102	0.455^*^	0.482^*^	0.097	0.577^**^
*P*	0.006	0.433	0.021	0.018	0.519	0.004

## Discussion

With the improvement of the quality of life and the aging of the population, the prevalence of LUTS and ED in men has been increased year-by-year (7), and the research on LUTS and ED has drawn more and more attention from a wide range of scholars. Seventy three percent of men aged 30–80 yr old suffer from LUTS and ED (15). At present, the clinical diagnosis of ED is mainly scored using the IIEF-5 followed by quantification, and these patients were asked about their sexual function in recent six months for comprehensive judgment (16). The diagnosis of LUTS is mainly evaluated using the IPSS questionnaire (17). Epidemiological studies with a large sample size (3, 18) showed that with the increase in age, the prevalence of LUTS and ED is significantly elevated. This study also confirmed that the prevalence of ED in patients with LUTS was significantly increased with the increase in age. In the survey of 8,000 men aged 30–80 years old, 72% of men with ED are combined with LUTS, whereas only 38% of men with normal erectile function suffer from LUTS (15). A number of surveys (19, 20) revealed that the prevalence of ED was significantly positively correlated with the level of LUTS after age and other risk factors are excluded. In this study, the prevalence of ED in patients with LUTS was 82.25%, and the prevalence of ED was positively correlated with the degree of LUTS. The higher the degree of LUTS was, the higher the prevalence of ED would be.

The erection of penis is a complex physiological process, involving the combined regulation of the nerve, blood vessels and internal secretion. In middle-aged and aged men, the vascular elasticity gradually became poor, which damages the organ filling, so the blood perfusion of penis corpus cavernosa is also reduced, thus affecting its erectile function (21). Some scholars believe that the correlation of ED with LUTS degree mainly depends on several factors (22): 1) The occurrence of LUTS and ED leads to metabolic syndrome and autonomic disorders; 2) the contents of nitric oxide and nitric oxide synthase in the pelvic cavity, prostate and penis are reduced; 3) the activities of endothelin and Rho kinase are increased; 4) atherosclerosis occurred in the bladder, prostate and penis blood vessels. Another study (23) speculates that the correlation between ED and LUTS degree may be because LUTS seriously affects the life of men and further affects their erectile function psychologically, thus leading to the occurrence of ED.

In addition to age, hypertension, diabetes mellitus and hyperlipidemia are also risk factors for ED (24). Some patients with hypertension, hypercholesterolemia and diabetes mellitus are often accompanied by vascular endothelial dysfunction, leading to atherosclerosis and ultimately resulting in ED (25). More and more studies have confirmed that ED can be used as a risk marker for cardiovascular disease (26, 27). Patients with diabetes mellitus are often complicated by ED prior to those with coronary heart disease, suggesting that this may be because diabetes mellitus injuries penile artery endothelium, thus leading to atherosclerosis, and with the prolonged course of the disease, aortic atherosclerosis finally occurs (28). This study also confirmed that the ED degree of patients was significantly related to the combination with hypertension, diabetes mellitus and coronary heart disease and the level of blood lipids. The levels of TC and LDL-C in patients with severe ED patients were significantly higher than those in patients with mild and moderate ED, indicating that the levels of serum TC and LDL-C are significantly positively correlated with ED prevalence. It was speculated that hyperlipidemia could impair vascular endothelial function of the penis, which led to the occurrence of atherosclerosis and blood vessel blockage, thus eventually resulting in the occurrence of ED. The number of corpus cavernosum endothelial cells and smooth muscles in white rabbits fed with high-fat diets (29). High Homocystinemia is an independent risk factor for arterial occlusive disease and cardiovascular and cerebrovascular disease, such as atherosclerosis (30). The level of serum Hcy in patients with ED is significantly higher than that in the normal group (31). Besides, the level of serum Hcy is negatively correlated with IIEF-5 score. This study also showed that the level of serum Hcy in patients with ED was increased with the increased degree of ED, indicating that ED prevalence is significantly positively correlated with serum Hcy level. It can be seen that the occurrence of ED has a significant correlation with atherosclerosis.

## Conclusion

The prevalence of ED of patients with LUTS is high, and the occurrence of ED is significantly correlated with the degree of LUTS. The older the patients are and the higher the LUTS degree is, the greater the risk of the combination with ED will be. Diabetes mellitus, hypertension, hyperlipidemia, coronary heart disease and other diseases are risk factors for ED, suggesting that the common pathology is the accompanying by atherosclerosis. Atherosclerosis can be used as a risk early warning for ED. In the clinical treatment of patients with LUTS, the ED-related issues should be considered at the same time. Improving patients’ arteriosclerosis can reduce and then delay the occurrence of ED.
